# Genoprevalence of cutavirus in benign and malignant intestinal and breast tissues

**DOI:** 10.1099/jgv.0.002184

**Published:** 2025-12-05

**Authors:** Irini M. Assimakopoulou, Ushanandini Mohanraj, Taina Sipponen, Anna Lepistö, Dalia Y. Kadry, Rana Hamdy, Mahmoud M. Kamel, Heba M. El-Batal, Ahmed S. Abdel-Moneim, Maria Söderlund-Venermo

**Affiliations:** 1 Department of Virology, University of Helsinki, Helsinki, Finland; 2 Gastroenterology, Abdominal Center, Helsinki University Hospital and University of Helsinki, Helsinki, Finland; 3 Department of Surgery, Helsinki University Hospital, Helsinki, Finland; 4 Applied Tumor Genomics, Research Programs Unit, University of Helsinki, Helsinki, Finland; 5 Clinical Pathology Department, National Cancer Institute, Cairo University, Cairo, Egypt; 6 Pediatric Oncology Department, National Cancer Institute, Cairo University, Cairo, Egypt; 7 Department of Medical Microbiology & Immunology, Cairo University, Giza, Egypt; 8 Department of Microbiology and Immunology, College of Medicine and Health Sciences, Sultan Qaboos University, Muscat, Oman

**Keywords:** breast cancer, colorectal cancer, cutavirus (CuV), human herpesviruses (HHV-6B, HHV-6A, HHV-7), inflammatory bowel disease (IBD), parvovirus

## Abstract

Cutavirus (CuV), the newest human protoparvovirus (PPV), has gained attention due to its significant association with cutaneous T-cell lymphoma (CTCL) and its precursor, parapsoriasis, whereas the other human PPVs, bufavirus and tusavirus, show no such link. Given this association, it is important to investigate the prevalence of CuV DNA in other tissues, particularly those affected by malignancy or inflammation. This study assessed, by multiplex quantitative PCR, the genoprevalences of all three PPVs in 427 fresh-frozen intestinal biopsies from inflammatory bowel disease (IBD), colorectal cancer, adenomas or healthy mucosa of 185 individuals, as well as in 94 formalin-fixed paraffin-embedded (FFPE) biopsies from malignant and non-malignant breast conditions of 85 patients. The study also compared the DNA prevalences of human herpesvirus (HHV)-6A, -6B and -7 in the breast tissues. CuV mRNA was assayed with reverse-transcription PCR, and corresponding FFPE sections underwent *in situ* hybridization. CuV DNA was detected in intestinal IBD or healthy mucosa from 6/185 (3.2%) subjects, but no CuV mRNA or *in situ* signals were detected. In breast biopsies, HHV-6B and HHV-7 DNAs were present in 20.3 and 5.1%, respectively, while all PPVs and HHV-6A were absent. Overall, CuV was absent in all 70 cancer tissues, underscoring its association with CTCL. The low CuV DNA loads and prevalences in intestinal and breast morbidities, and lack of activity, suggest that CuV is unlikely to play a role in these malignancies or inflammatory conditions. In contrast, HHV-6B may be more relevant to breast pathology, even though it is also widely detected in healthy tissues. Nevertheless, our study provides insight into persistent DNA viruses implicated in cancer and highlights their occurrence across various disease manifestations, laying a foundation for future studies.

## Data Availability

All Sequences submitted to GenBank; PQ553262–PQ553268. Requests should be submitted to the corresponding author, Irini M. Assimakopoulou (irini.assimakopoulou@helsinki.fi).

## Introduction

In 2012–2016, three new parvoviruses of the *Protoparvovirus* genus, named bufavirus (BuV), tusavirus (TuV) and cutavirus (CuV), were by metagenomics discovered in stools [1,3]. CuV has since been associated with cutaneous T-cell lymphoma (CTCL) and its precursor, parapsoriasis en plaques (PP), a chronic inflammatory condition in skin [3,6]. Recent studies have shown that in individuals with CTCL or PP, CuV remains persistent in skin for years, actively expresses mRNA, spreads via circulating immune cells within the body and is excreted to the environment in stools as potentially infectious virions [4,6]. CuV-specific seropositivity does not seem to hamper the active persistence of this virus.

Inflammatory bowel disease (IBD) is a long-term inflammatory condition that affects the digestive tract and is categorized as either Crohn’s disease or ulcerative colitis (UC) [7]. The cause of IBD is unknown, but it is considered the result of an inappropriate immune response against environmental factors, including luminal and microbial antigens in genetically susceptible hosts [8]. Associations between other persistent parvovirus infections such as parvovirus B19 (B19V) or human bocavirus (HBoV) and IBD or gastrointestinal tumours have been suggested, but their causative role has not been verified [9,13]. Further, several persistent DNA viruses have been implicated in cancer, including human herpesviruses (HHVs), papillomaviruses and polyomaviruses [14]. The nine HHVs are globally ubiquitous pathogens that persist lifelong and can reactivate, sometimes causing severe diseases. However, whether HHV-6 or -7 has direct roles – or can act as contributory factors – in tumourigenesis is less clear; their prevalence and role in breast cancer have rarely been studied [15,16].

Hence, the aim of our current study was to elucidate the DNA prevalence of recently discovered human protoparvoviruses (PPVs), BuV, TuV and CuV, in intestinal and breast biopsy specimens from patients with IBD, colorectal cancer or malignant or non-malignant breast conditions. Additionally, as all PPV PCRs were negative in the breast samples, we also investigated the DNA prevalence of the more common HHV-6A, -6B and -7, since they are rarely examined in breast biopsy specimens.

## Methods

### Patients and clinical specimens

#### Gut cohort

In total, 427 fresh-frozen intestinal specimens from 185 individuals (aged 20–86 years; mean 51; median 52.3) were biopsied at the Helsinki University Hospital (Helsinki, Finland) and included in our study. These samples had been previously studied for parvovirus B19 and HBoV infections [13]. Samples were immediately immersed in RNAlater at 4 °C and subsequently preserved at −20 °C until nucleic-acid extraction. Of the 185 individuals presenting with malignancy (*N*=16), active (*N*=42) or inactive (*N*=33) UC, or adenoma (*N*=39), 129 provided paired mucosa of the disease-affected colon and adjacent healthy colon or ileum segments or both. The remaining 55 individuals were by histopathological examination considered healthy, providing only healthy colon or ileum biopsies.

Of these biopsied individuals, only a total of 13 serum samples were available, from 4 patients with inactive UC, 7 with active UC and 2 with adenoma. For *in situ* hybridization, 11 formalin-fixed paraffin-embedded (FFPE) samples were additionally obtained from Helsinki Biobank from 5 CuV DNA-positive and, as negative controls, 2 CuV PCR-negative individuals, all from the same intestinal regions as the fresh-frozen RNAlater-stored samples.

#### Breast cohort

In total, 94 FFPE tumour tissue specimens from 85 patients (aged 27–82 years; mean 52; median 50.5) were collected from the Baheya Centre for Early Detection and Treatment of Breast Cancer and National Research Centre (Cairo, Egypt) between 2022 and 2023. Patients were diagnosed with breast cancer (*n*=53), benign breast conditions (*n*=28), other benign conditions (*n*=3) or prostate cancer (*n*=1), as detailed in Table S3, available in the online Supplementary Material. Tissue slices were cut from FFPE tissue blocks and stored as rolls in microtubes at room temperature until DNA extraction.

### Ethics

The study was approved by the Ethics Committee of the HUS Helsinki University Hospital (Decision number 553/E6/01, amendments 15 July 2014 and 2322/2024) and the Baheya Research Ethics Committee (Decision number 202302200006), and informed consent was obtained from all individuals.

### Methods

All methods mentioned here, in short, are further described in File S1.

#### DNA and RNA extraction, PCR and EIA

Total DNA and RNA were separately extracted, PPV and HHV DNA detected and quantified by multiplex quantitative PCRs (qPCRs), spliced CuV mRNA detected and quantified by reverse-transcription (RT) PCR (Table S2) and PPV IgG and IgM measured by in-house enzyme immunoassays (EIAs), as previously published [4,6, 17,20]. In addition, the CuV DNA-positive samples, identified by PPV multiplex-qPCR screening (targeting a short 91 bp VP2 region), were subsequently selected for amplification of also longer (>500 bp) VP1 and VP2 fragments for phylogenetic analysis; all primers and probes are described in Table S2, and the resulting sequences were submitted to GenBank (PQ553262–PQ553268) [2]. All samples also underwent human *RNaseP* qPCR or *RPII* RT-PCR to control for cell DNA or RNA integrity, respectively.

#### RNAscope *in situ* hybridization

RNAscope ISH (RISH) technology (ACD, Newark, CA) was applied on the seven gut FFPE biopsies with Probe-V-CuV-NS1, with positive and negative controls [6].

### Statistical analysis

The Fisher’s exact test was used for comparison of virus prevalence in different disease groups. *P* values<0.05 were considered statistically significant.

## Results

### PPV DNA prevalence in intestinal biopsy specimens of the gut cohort

Only 7 of 427 (1.6%) intestinal specimens from 6/185 (3.2%) individuals harboured CuV DNA: 1/42 (2.4%) with active UC, 1/33 (3%) with inactive UC, 1/39 (2.6%) with adenoma and 3/55 (5.5%) from healthy subjects, in various intestinal sites (Table 1). Moreover, only one of these six individuals exhibited CuV DNA in both biopsies, while otherwise mostly in the healthy segment. Only one CuV DNA-positive specimen (IBD 45) showed histopathological signs of disease: a hyperplastic polyp. All CuV DNA loads were low, <10^5^ CuV copies per one million cells (cpm) (Table 1). Interestingly, CuV DNA was not detected in any of the 16 malignant gut tissues. The prevalence of CuV DNA in patients of each disease group separately, or together, was somewhat lower than that among the healthy individuals, but without reaching statistical significance. All specimens were human *RNaseP*-PCR positive but BuV- and TuV-DNA negative. The amplification plot and quantification cycles of the CuV plasmid standards are shown in Fig. S1A and Table S1.

**Table 1.** Characteristics of the six individuals with CuV PCR-positive biopsies in the gut cohort

Patient ID	Diagnosis	Tissue histopathology	CuV DNA*[Table-fn T1_FN1]	CuV mRNA	CuV IgG (OD)
IBD 10	Healthy	Healthy sigmoid colon	1.1×10^2^	na	na
		Healthy ileum	Neg.	nd	
IBD 24	Healthy	Healthy ileum	3.3×10^1^	na	na
		Healthy sigmoid colon	5×10^2^	na	
IBD 45	Inactive UC	Healthy ileum	Neg.	nd	2.8
		Sigmoid colon with hyperplastic polyp	7.7×10^2^	Neg.	
IBD 107	Healthy	Healthy ileum	7.1×10^4^	Neg.	na
		Healthy colon	Neg.	nd	
IBD 119	Adenoma	Ascending colon with polyps	Neg.	nd	na
		Healthy descending colon	8.2×10^0^	Neg.	
IBD 155	Active UC	Severely inflamed descending colon	Neg.	nd	na
		Healthy ileum	2.3×10^2^	Neg.	

* Copies per one million cells, based on the single-gene *RNase* P copies; mean of duplicate wells. BuV and TuV PCRs were negative. Patients aged 25–79 years, mean 54.7, median 57.7. na, not enough sample available; nd, not done. In all samples, *RNaseP* DNA copies per µl ranged between 3.6×104 and 1.5×105 and *RPII* RNA copies between 4.9×101 and 5.4×102, exhibiting a melting temperature of 84.5 °C (Fig. S1B).

### CuV mRNA and RISH of intestinal biopsies

To look for potential virus activity, we searched for mRNA by RT-PCR in the four available CuV DNA-positive fresh-frozen RNAlater-submerged intestinal biopsies, but despite all being human *RPII* mRNA positive (Fig. S1B), they were CuV mRNA negative (Table 1). To look for viral cell tropism, we applied RISH to all five available CuV PCR-positive FFPE intestinal biopsies, but none showed CuV-specific *in situ* signals, likely due to the low viral loads (Fig. S2).

### CuV DNA sequence analysis

**Fig. 1.**
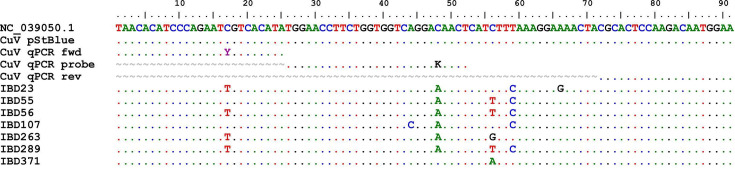
Alignment of 91 nt CuV sequences derived from CuV DNA-positive samples in the gut cohort. IBD23 (healthy sigmoid colon, patient IBD 10), IBD55 (healthy ileum, patient IBD 24), IBD56 (healthy sigmoid colon, patient IBD 24), IBD107 (sigmoid colon with hyperplastic polyp, patient IBD 45), IBD263 (healthy ileum, patient IBD 107), IBD289 (healthy descending colon, patient IBD 119) and IBD371 (healthy ileum, patient IBD 155). CuV reference sequence NC_039050.1 (nt 4,245–4,335). CuV pStBlue was the control plasmid in qPCR. Dot (.), identical nucleotides; tilde (∼), lacking nucleotides; Y=C or T; K=G or T.

**Fig. 2.**
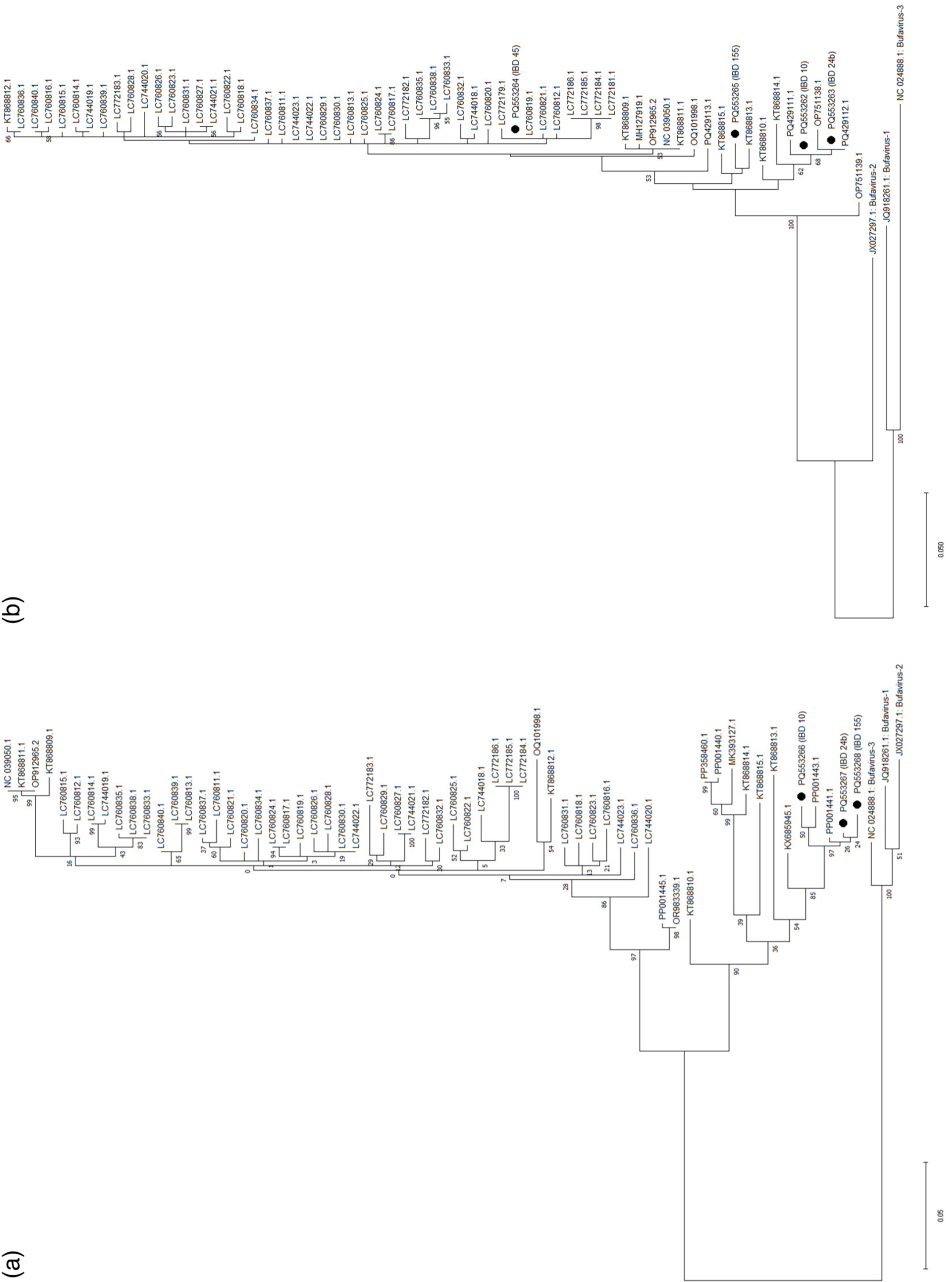
Maximum likelihood phylogenetic tree of (a) *VP1* and (b) *VP2* regions from CuV DNA-positive gut samples. (**a**) 468 nt amplicons in the *VP1* region (NC_039050.1: nt 2,048–2,515) and (b) 460 nt amplicons in the *VP2* region (NC_039050.1: nt 3,868–4,323). The *VP1* analysis included 65 nucleotide sequences, and *VP2* included 66. BuVs 1–3 were included as outgroups. Sequences annotated as filled circles are from this study. The percentage of trees in which the associated taxa clustered together is shown next to the branches. The tree is drawn to scale, with branch lengths measured in the number of substitutions per site. IBD 10 (healthy sigmoid colon); IBD 24 (healthy sigmoid colon); IBD 45 (sigmoid colon with hyperplastic polyp); IBD 155 (healthy ileum); all IDs refer to patients.

The seven 91 nt CuV *VP2*-amplicon sequences from six patients were similar but differed between individuals (Fig. 1). For four patients, longer, ~500 nt, CuV *VP1* and *VP2* sequences were successfully obtained and aligned with sequences in GenBank. Based on phylogeny, three longer *VP1* sequences clustered together, whereas the corresponding three *VP2* sequences clustered less closely (Fig. 2). The fourth VP2 sequence of IBD 45 was closer to the Japanese LC sequences. The BuV2-*VP2* outgroup is situated closer to the CuV sequences, while the CuV-*VP1* sequences formed a separate clade. We were not able to sequence longer fragments. The tree with the highest log likelihood for *VP1* (−3845.91) is shown in Fig. 2(a) and for *VP2* (−2432.05) in Fig. 2(b).

### Serology of the gut cohort

Only 1/13 (7.7%) serum samples was CuV IgG positive with OD 2.4 (IBD 45 with inactive UC), who also harboured CuV DNA in gut tissue. This individual was further both CuV IgM and CuV DNA negative in serum, indicating prior immunity. We found two additional individuals with elevated CuV and BuV2 IgG, with low absorbances, but the results were regarded as inconclusive based on the competitive blocking assay [21]. All sera were IgG negative for BuV1, 3 and TuV. No sera were available from the breast cohort.

### PPV and HHV DNA in malignant and benign tissue specimens of the breast cohort

No PPV DNA was detected in 94 FFPE breast and prostate tumour tissue specimens from 85 patients. Of the 79/85 patients with breast specimens still available for herpesvirus PCR, 16 (20.3%) harboured HHV-6B DNA and 4 (5.1%) HHV-7 DNA, while none were positive for HHV-6A DNA. In total, HHVs were found in tissues of 20 out of 79 (25.3%) patients (Table 2).

**Table 2.** Characteristics of the breast cohort and presence of viral DNA

Disease group*	Patient, *N*	PPV+ patient, *N* (%)	HHV-6A+ patient, *N* (%)	HHV-6B+ patient, *N* (%)	HHV-7+ patient, *N* (%)
Breast cancer	53	0	0	9 (17)	1 (1.9)
Benign breast conditions	28	0	0	7 (25)	2 (7.1)
Prostate cancer	1	0	0	0	1
Other benign conditions (ovary, skin, thyroid)	3	0	0	0	0
All	79	0 (0)	0 (0)	16 (20.3)	4 (5.1)

*Precise clinical diagnoses are found in Table S3. *RNaseP* copies per µl ranged between 4×101 and 2.2×104.

The HHV-6B DNA loads ranged between 2.1×10^2^ and 6.7×10^6^ cpm, while the HHV-7 DNA loads were 1.1×10^2^ to 1.6×10^3^ cpm. The occurrence of HHV DNAs, separately or together, in the malignant and benign disease groups was compared with Fisher’s exact test, but the differences did not reach statistical significance in any combinations. All tissue samples contained human DNA as extraction control.

## Discussion

Investigating the prevalence of newly discovered viruses across different tissue specimens is crucial to determine their association with disease and where they persist [22]. Given the association of CuV with CTCL and its precursor PP [3,6,23], it is necessary to investigate its prevalence, persistence, activity and possible tissue-distribution sites in other malignant, inflamed or other tissue types.

This is the first study to elucidate the presence of CuV, BuV and TuV DNA in intestinal and breast tissues. The overall intestinal CuV DNA prevalence among all 185 patients in the gut cohort was low (3.2%), and no CuV DNA was detected in breast tissues of the 85 patients in the breast cohort. Our genoprevalences are comparable with those previously reported in skin biopsies of transplant patients (2.9%), melanomas (1.1%), head-and-neck tumours (4%) and healthy individuals (0%), which correlate to the previously reported low CuV IgG seroprevalence among healthy adults (0–7%) [4,21, 26,29], and much lower than those in skin from patients with CTCL (especially subtype mycosis fungoides; MF) (8.5–38%) and its precursor PP (38–66.7%) [3,6,23].

Notably, we did not detect CuV DNA in any malignant tumours from colorectal, breast or prostate cancer patients, even if some parallel healthy segments did harbour the virus, emphasizing its specific and significant association with the skin lymphoma.

The 91 bp CuV sequences obtained from six patients were not identical, ruling against plasmid or cross-contamination. Phylogenetic analysis of the longer CuV DNA sequences from four isolates in the *VP1* region showed close clustering with CuV strains from PP patients in Finland (PP001441.1 and PP001443.1) [6]. In contrast, the VP2 sequences displayed a slightly different pattern, with one isolate clustering with strains from France and Botswana (KT868815.1 and KT868813.1, respectively) and another isolate with strains from Japan [3,25, 30].

Our previous findings indicate that persistent CuV is actively transcribing in CTCL and PP tissues, as spliced viral mRNA was detected in the skin of these patients [6]. Expression of mRNA indicates active viral gene transcription in the nucleus, potentially impacting the cellular and tumour microenvironment. However, in this study, CuV mRNA was not detected in the four available CuV DNA-positive fresh-frozen tissues, raising a hypothesis that CuV activity may be specific to CTCL-MF and PP tissues. Our results thus suggest that CuV, in non-CTCL-MF tissues, persists in a dormant state, a characteristic commonly observed with other human parvoviruses [13,22]. Further studies involving diverse tissue types are necessary to confirm these observations.

No CuV-specific signals were detected by RISH in the corresponding FFPE biopsies from the five patients in the gut cohort with CuV PCR-positive fresh-frozen intestinal biopsies, possibly due to the lower sensitivity of the RISH assay compared to that of the qPCR assay. The hospital does not store serum samples from endoscopy patients; however, we were able to obtain sera from 13 patients, one of whom happened to carry CuV DNA in his intestinal mucosa. In all, 1/13 (7.7%) exhibited CuV IgG, whereas all were IgM negative, indicating a lack of acute infections. Given the limited number of serum samples, chance may have contributed to the observed seroprevalence. In previous studies, the CuV IgG prevalence has been observed to be similar among healthy adults (0–7%) compared to the slightly higher seroprevalence in CTCL and PP patients (9.5–33.3%), <65 years of age [4,6, 19, 21, 27]. Of note, differences in age and ethnicity may affect the seroprevalence.

In an earlier study of the gut cohort, parvovirus B19 DNA persisted frequently in the intestinal mucosa of patients with colorectal cancer (50%), UC (47%), adenoma (31%) and healthy controls (27%) [13]. In our present study of the same cohort, the frequency of CuV DNA persistence was much lower than that of B19V, revealing respective prevalence rates of 0, 2.7, 2.6 and 5.5%, which are closer to the genoprevalence of HBoV DNAs in the same cohort [13]. Interestingly, similarly to HBoV DNA that was detected in only histopathologically healthy areas of the mucosa, CuV DNA was also found in the healthy or inactive mucosal regions. B19V DNA, however, persisted in both healthy and diseased tissues, but it was significantly more prevalent in the healthy colonic segments of UC patients than in the diseased ones [13]. In both the previous and the current studies, the differences in virus occurrence between each disease group and the healthy control patients did not reach statistical significance. Of the seven CuV DNA-positive samples, two (IBD 45; sigmoid colon, and IBD 155; ileum) also carried B19V DNA.

The B19V genoprevalence across various tissues ranges from 20 to 60% [31,34], whereas CuV shows a broader variability, ranging from 0 to 67%, depending on the underlying disease, although some estimates are based on relatively small sampling sizes [3,6,23, 24, 26, 28].

In addition to PPV DNA, the breast cohort was also tested for HHV-6 and -7, which have, similar to B19V, been shown to persist in tissues [38]. The results showed a higher prevalence of HHV-6B at 20.3%, compared to HHV-7 at 5.1% and HHV-6A at 0%. This is the first large-scale study to search for HHV-6 and HHV-7 in breast specimens, including both malignant and benign cases. Only a few studies have explored the relationship between breast cancer and HHV-6, and none of HHV-7 [16,39]; one examining if inherited chromosomally integrated HHV-6 is a breast cancer risk factor but without any supporting evidence, and the other comprising ten breast cancer control specimens, all negative for HHV-6.

In our study, the prevalence rates of both HHV-6B and HHV-7 DNA in breast tissues were slightly lower in breast cancer patients compared to those of benign breast conditions, ruling against oncogenicity, but the differences were not significant. HHV-6 and HHV-7 are pathogens that persist for life in our tissues and can reactivate. That HHV-6A and HHV-6B have been detected in various cancers does not provide direct evidence of them being oncogenic; instead, they are suggested to indirectly promote tumour cell growth alongside other viruses [16]. We did not find any tissue samples with both CuV and HHV DNAs in the same tissue.

The breast cohort comprised only FFPE samples, which may have led to increased DNA fragmentation and, consequently, lower PCR sensitivity and lower human *RNase P* loads compared to the fresh gut samples; however, it is comparable to the FFPE samples of CTCL and PP skin [4,5]. Moreover, although RISH has been successful in our past CTCL and PP studies [5,6], the CuV DNA loads were too low for any signals to be detected in this study. Despite these factors, the patient materials from both cohorts are valuable, particularly as we report novel findings on CuV, a lymphoma-linked virus, in malignant or inflamed and other benign intestinal and breast tissues, as well as on the pathogenic herpesviruses HHV-6 and HHV-7 in large-scale breast specimens. While the links between protoparvo- and herpesviruses and diseases remain inconclusive, screening for these DNA viruses – which persist and are implicated in cancer – provides valuable insights into the prevalence and persistence of them, laying the foundation for further studies.

## Supplementary material

10.1099/jgv.0.002184 Uncited Supplementary Material 1.

## Abbreviations

BuV: bufavirus
cpm: copies per one million cells
CTCL: cutaneous T-cell lymphoma
CuV: cutavirus
EIA: enzyme immunoassay
FFPE: formalin-fixed paraffin-embedded
HBoV: human bocavirus
HHV: human herpesvirus
IBD: inflammatory bowel disease
MF: mycosis fungoides
PP: parapsoriasis en plaques
PPV: protoparvovirus
qPCR: quantitative PCR
RISH: RNAscope ISH
RT-PCR: reverse-transcription PCR
TuV: tusavirus
UC: ulcerative colitis
