# Supplementary material for: Genoprevalence of cutavirus in benign and malignant intestinal and breast tissues
Source: J Gen Virol. 2025 Dec 5;106(12):002184. doi: 10.1099/jgv.0.002184 (PMC12680079; doi:10.1099/jgv.0.002184)
Supplement: Uncited Supplementary Material 1. [file jgv-106-02184-s001.pdf]

Supplementary Methods

DNA and RNA extraction

Total DNA was extracted from all fresh-frozen intestinal biopsy specimens with the QIAamp DNA mini kit (Qiagen, Germany) according to the manufacturer's protocol. DNA extractions from FFPE samples were performed with the QIAamp DNA FFPE tissue kit (Qiagen, Germany), according to the manufacturer's instructions with some modifications. Due to the thick FFPE slices, ranging from 10 µm to 0.2 mm, paraffin removal was performed three times (30 min, 60°C, 700 RPM), then 80 µl of proteinase K together with carrier-RNA (Qiagen, Germany; final concentration 2.5x10-3 µg/µL) was added after the 90°C incubation step. DNA extracts of paraffin slices exceeding 10 µm in thickness were analysed both directly and as 1:10 dilutions in ATE buffer. DNA was eluted in 60 µl buffer. RNA was extracted with the QIAamp RNA Mini Kit (Qiagen, Germany), including a DNase treatment, and RNA was eluted in 20 µl elution buffer. All DNA and RNA extracts were stored at −20°C, and reverse-transcription (RT)-PCR was completed within a few hours of extraction, as previously reported for CTCL and PP tissues [1].

Protoparvovirus (PPV) DNA multiplex qPCRs

PPV DNAs were detected and quantified in the gut and breast cohorts, by an in-house real-time multiplex quantitative PCR (qPCR) targeting the VP2 region of CuV (91 bp) and TuV (118 bp) and the NS1 region of BuV (126 bp), as previously reported [2]. All positives were confirmed by cloning and sequencing, as previously published [1,2]. The PPV qPCR detected CuV DNA down to 1 copy/µL (Figure S1A and Table S1). The primers, probes and performance values for the PPV qPCR, along with the criteria used to select the most optimal target regions, are published [2]. DNA quality and human-cell quantity in all samples were evaluated with the reference human one-copy gene *RNaseP* qPCR, as previously reported [3,4,5]. Ten-fold diluted plasmids containing the target amplicons (viral or *RNase P* targets) served as standards and positive controls (Figure S1A, Table S1), and viral DNA copies were normalized to one million human cells [1,2]. Water served as negative controls and the samples were tested in duplicates in all PCRs. All primers and probes are shown in Table S2.

CuV DNA-sequence analysis

Following qPCR screening (91-bp amplicon; VP2 target), the CuV-positive samples were used to amplify longer (>500 bp) VP1 and VP2 fragments. Longer DNA fragments of 502 - 569 nucleotides (nt) were sequenced from seven CuV-DNA positive samples either with primers CuV-RT-VP1-T1 fwd and CuV-RT-VP1 rev for the VP1 region, or with primers CuV VP2 583 fwd and CuV rev for the VP2 region (Table S2), as previously reported [1,2]. The sequences were submitted to GenBank (PQ553262-PQ553268). The shorter VP2 target (91 bp) detected in the initial qPCR screening is located within the longer VP2 fragment amplified in the subsequent step. The phylogenetic tree was inferred with the Maximum Likelihood method and the Tamura-Nei model, with a bootstrap test of phylogeny performed using 1000 bootstrap replications [8]. Initial tree(s) for the heuristic search were obtained automatically by applying Neighbor-Join and BioNJ algorithms to a matrix of pairwise distances estimated using the Tamura-Nei model, and then selecting the topology with superior log likelihood value. Evolutionary analyses were conducted in MEGA11 [9].

Cutavirus mRNA RT-PCR

A CuV-specific two-step semi-quantitative RT-PCR was performed to detect and quantify spliced CuV mRNA, exactly as previously reported [1,6]. In short, RT was performed for 1 h at 37°C with M-MLV Reverse Transcriptase (Life Technologies, USA) and random hexamers (Promega, USA), followed by a SYBR Green-based CuV qPCR reaction in Maxima SYBR Green qPCR Master Mix (Thermo Scientific, USA) with primers CuV-RT-VP1-T1 Fwd and CuV-RT-VP1 Rev, amplifying 210-nt long mRNA transcripts, spliced at position 1519–1884 (Table S2) [1]. A plasmid with cloned cDNA from spliced mRNA of a CuV PCR-positive skin tissue, served as standard for the CuV-mRNA RT-PCR [1]. The mRNA quality was verified with the human gene *RPII* mRNA RT-PCR (Figure S1B) [1]. The spliced target, DNase treatment and a no-RT control, ruled out DNA contaminations in RT-PCR.

HHV-6A, HHV-6B, and HHV-7 DNA qPCRs

HHV-6A, HHV-6B, and HHV-7 DNAs were searched for and quantified in biopsy samples from the breast cohort (Table S3: 88 samples from 79/85 patients were still available for HHV PCR), by HERQ-9 multiplex qPCR for the three HHVs, exactly as previously published, with primers, probes, sensitivity and

reproducibility data (Table S2) [7]. Ten-fold diluted plasmids containing the target amplicons (viral or *RNase P* targets) served as standards and positive controls, and viral DNA copies were normalized to one million human cells. Water served as negative controls and the samples were tested in duplicates. As our primary focus was on CuV, we did not perform HHV mRNA RT-PCRs or RISH.

RNAscope in situ hybridization (RISH)

RNAscope ISH (RISH) technology (Advanced Cell Diagnostics [ACD], Newark, CA) was applied to identify the target cells of CuV in FFPE gut tissues, by localizing the CuV DNA/RNA in PCR-positive 5-µm-thick FFPE tissue sections mounted on SuperFrost Plus glass slides. Viral nucleic acid was detected with double-Z oligonucleotide antisense-probes targeting the CuV *NS1* gene (Probe-V-CuV-NS1, 2023 – 3562 bp; GenBank: NC_039050.1), as previously reported [1]. The hybridized probe signal was amplified with RNAscope 2.5 HD Reagent Kit-RED (ACD), according to the manufacturer's protocol. Probes targeting the human housekeeping gene *PPIB* and the bacterial gene *dapB* (ACD) served as positive and negative technical controls, respectively. CuV RISH of controls and a representative sample are shown in Figure S2. Stained tissue sections were scanned with the 3DHISTECH Pannoramic 250 FLASH II digital slide scanner at the Genome Biology Unit, supported by HiLIFE and the Faculty of Medicine at the University of Helsinki and Biocenter Finland. Bright-field images were generated and analysed with the CaseViewer software (3DHISTECH).

Serology

A total of 13 serum samples were available from the gut cohort (age range: 20.4–61.6 years, with a mean of 44.5 and a median of 52.3) and analysed by BuV1-3-TuV-CuV IgG and IgM enzyme immunoassays (EIA), with respective biotinylated VP2 baculovirus-produced virus-like particles (VLPs) as antigens [10,11]. Due to cross reactivity between the PPVs, all samples with OD >0.1 were re-analysed after cross-blocking with homo- and heterotypic VLPs, as previously reported [10,11]. A sample was classified as IgG positive if it demonstrated almost complete inhibition in homotypic blocking but no or minor inhibition in heterotypic blocking.

Supplementary Results as Figures and Tables:

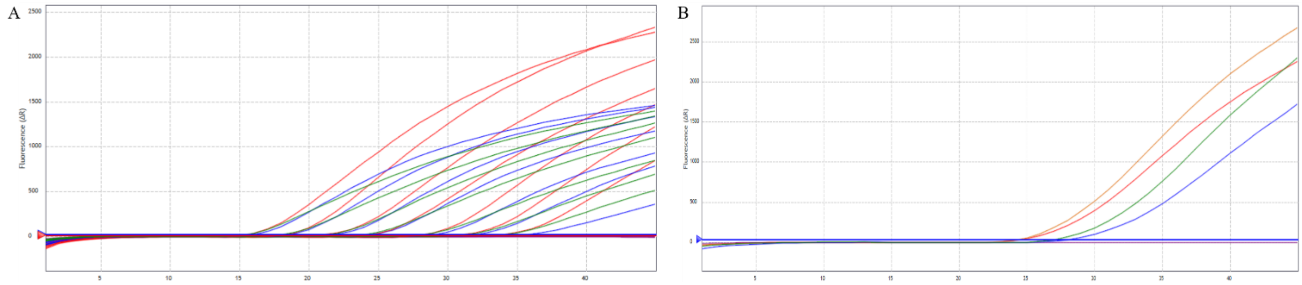

Figure S1. A) Amplification plot for PPV quantitative PCR. Curves in different colors represent serial dilutions of CuV (red), BuV (blue), and TuV (green) plasmid standards, along with a no-template (water) control (purple). Fluorescence intensity (ΔR, y-axis) is plotted against cycle number (x-axis). No amplification was observed in the no-template control. Cutoffs in the same colours shown as straight lines. **B) Amplification plot for *RPII* RT-PCR.** Colored curves represent *RPII* mRNA levels in patient samples from the gut cohort (IBD 45, red; IBD 107, orange; IBD 119, blue; IBD 155, green) and a no-template (water) control (purple). Fluorescence intensity (ΔR, y-axis) is plotted against cycle number (x-axis). *RPII* showed a consistent melting temperature of 84.5 °C across all samples and *RPII* dilutions. No amplification was observed in the no-template control. Cutoffs in same colors are shown as straight lines.

Table S1. Quantification cycle (Cq) values for viral qPCRs.

Copies/µL	Target Fluorescent Dye (Cq) for PPVs	Target Fluorescent Dye (Cq) for HHVs
10^0	JOE (34.1); FAM (36.7); TEXASRD (34.2)	Not included
10^1	JOE (31.3); FAM (32.7); TEXASRD (31.4)	JOE (33.8); FAM (33.7); TEXASRD (31.9)
10^2	JOE (28.5); FAM (29.7); TEXASRD (28.5)	JOE (29.3); FAM (29.5); TEXASRD (28.6)
10^3	JOE (24.5); FAM (25.3); TEXASRD (24.1)	JOE (26.3); FAM (26.3); TEXASRD (25.4)
10^4	JOE (21.3); FAM (22.5); TEXASRD (21.4)	JOE (22.6); FAM (23.0); TEXASRD (21.9)
10^5	JOE (18.5); FAM (19.2); TEXASRD (18.2)	JOE (19.4); FAM (19.5); TEXASRD (18.6)
10^6	JOE (18.5); FAM (16.6); TEXASRD (18.2)	JOE (15.8); FAM (16.2); TEXASRD (15.4)
H_2O	JOE (No Cq); FAM (No Cq); TEXASRD (No Cq)	JOE (No Cq); FAM (No Cq); TEXASRD (No Cq)

Quantification cycle (Cq) values for serial dilutions of plasmid standards of CuV, BuV, and TuV (PPVs) and HHV-6B, HHV-6A, and HHV-7 (HHVs), detected with JOE, FAM, and TEXASRD fluorescent dyes, respectively, along with a water control. Sensitivity (LOD) and inter/intra-assay variabilities for protoparvoviruses can be found in previous reports [2,7].

Table S2. Primers and probes used in this study.

Oligo name	Sequence (5'-3')	Amplicon bp (nt)	Reference
CuV VP2 Fwd Primer	TAACACATCCCAGAATYGTCACATA	91 (4245–4335)	[1,2]
CuV VP2 Rev Primer	TTCCATTGTCTTGGAGTGCG		
CuV VP2 Probe	JOE-AGTTKTCCTGACCACCAGAAGGTTCCA-BHQ1		
BuV NS1 Fwd Primer	ACAGTGTAGACAGTGGATTCAAACTT	126 (705–830)	
BuV NS1 Rev Primer	GTTGTGGTTGGATTGTGGTTAGTTC		
BuV NS1 Probe	FAM-CGGAAGAGATTTTGACAGTGCYTAGCAA-BHQ1		
TuV VP2 Fwd Primer	CCAGAAAGCCGTATCACCAT	118 (3085–3202)	
TuV VP2 Rev Primer	AACCAAGTGTTTCTGATCTTATTGCT		
TuV VP2 Probe	TxRd-ACACCAACAATCAACTGCCATACACACC-BHQ2		
CuV RT VP1 T1 Fwd Primer	AGGATGCCAGCTATTAGAAAAGC	210[a] (1494–1516) 572[b] (1494–2065)	[1]
CuV RT VP1 Rev Primer	GACGAAGACTTCATCAAAGCAAC		
CuV VP2 583 Fwd Primer	TCAACAACTGAAGGMACCAGACTAAC	583[c] (3753–4335)	[2]
CuV VP2 Rev Primer	TTCCATTGTCTTGGAGTGCG		
RPII RT Fwd Primer	GCACCACGTCCAATGACAT	632 (838–1469)	[6]
RPII RT Rev Primer	GTGCGGCTGCTTCCATAA		
RPII RT Probe	FAM-TACCACGTCATCTCCTTTGATGGCTCCTA-BHQ1		
RNaseP Fwd Primer	GAGGGAAGCTCATCAGTGGGG	84 (9–92)	[3,4,5]
RNaseP Rev Primer	CCCTAGTCTCAGACCTTCCCAAG		
RNaseP Probe	FAM-AGTGCGTCCTGTCACTCCACTC-TAMRA		
HHV6A U90 Fwd Primer	CGGCCTCCAGAGTTGTAA	76 (133969–133894)	[7]
HHV6A U90 Rev Primer	TGTCCCTTCAACTACTGAATC		
HHV6A U90 LNA Probe A1	FAM-AC[+A]T[+G]TTGC[+T]A[+G]AAA[+G][+A]CT-BHQ1		
HHV6A U90 LNA Probe A2	FAM-AC[+A]T[+G]TTGC[+T]A[+C]AAA[+G][+A]CT-BHQ1		
HHV6B U90 Fwd Primer	TTTGACAGGAGTTGCTGAG	83 (136176–136258)	
HHV6B U90 Rev Primer	GGATTCAGGAAAAAGGTTCTAA		
HHV6B U90 Probe	JOE-AGGAAGCGTTTCGGTACACTTGGAG-BHQ1		
HHV7 U57 Fwd Primer	CTCGCAGATTGCTTGTTG	159 (88332–88490)	
HHV7 U57 Rev Primer	GCATACACCAACCCTACTGTAA		
HHV7 U57 Probe	TxRd-TTAGGCATCACGTTGGCATTG-BHQ2		

Two probes were used simultaneously for HHV-6A detection. [a]Spliced CuV mRNA transcript. [b]CuV genomic DNA; longer VP1 fragment. [c]CuV genomic DNA; longer VP2 fragment. RT, reverse transcriptase; Fwd, forward; Rev, reverse; LNA, locked nucleic acids; bp, base pairs; nt, nucleotides; Y= C + T; K = G + T.

Table S3. Precise diagnoses of the breast cohort and presence of HHV DNA.

**Diagnosis/ Tissue Histopathology**	**Patient ID**
Invasive duct carcinoma, grade I	BR36, *BR75*^{6B}
Invasive duct carcinoma, grade II	BR2a, BR2b, BR4a, BR4b, BR9a*, BR9b, BR14, BR18*, BR30, **BR31**, *BR43*^{7}, BR44*, *BR45*^{6B}, BR62, BR63*, BR67, BR69, ***BR85***^{6B}, *BR86*^{6B}, *BR92*^{6B}, BR93, BR95
Invasive duct carcinoma, grade III	BR10, BR20, BR50, BR52, BR60, BR68, *BR73*^{6B}, BR74, BR83
Multifocal Invasive duct carcinoma, grade II	*BR13*^{7}, BR21, BR54
Metastatic duct carcinoma grade III	BR37
Bilateral invasive duct carcinoma, grade III	BR22a, *BR22b*^{6B}
Multicentric invasive duct carcinoma, grade I	BR61
Multicentric invasive duct carcinoma, grade II	BR38, BR49, BR84*
Multicentric invasive duct carcinoma, grade III	BR24
Ductal carcinoma in situ with focal stromal microinvasion	BR19
Invasive lobular carcinoma grade II	BR26, *BR35*^{6B}, BR53, BR76, BR91*, BR94
Invasive micropapillary carcinoma, grade II	**BR31**
Invasive mucinous carcinoma, grade II	BR47, ***BR85***^{6B}
Mucinous carcinoma grade I	BR55
Metastatic poorly differentiated carcinoma	BR59
Metaplastic carcinoma, adenosquamous subtype, grade III	BR64a, BR64b
Invasive breast carcinoma with medullary pattern, grade III	BR1a, BR1b
Borderline phyllodes tumor	**BR78**
Prostatic acinar adenocarcinoma, grade III	*BR15*^{7}
Microcalcifications	BR3a^{6B}, BR3b, **BR11, BR17, BR89**
Fibrocystic disease	*BR5a*^{6B}, BR5b, **BR11,** *BR71*^{6B}, **BR78**
Intraduct papilloma	**BR8, BR23,** *BR32a*^{6B}, BR32b, BR58
Focal usual ductal hyperplasia	**BR11, BR17**
Duct ectasia	**BR8, BR23, BR66**
Benign phyllodes tumor	*BR12*^{7}, BR72
Fibroadenoma	*BR16*^{6B}, BR33, BR39, BR40, BR77, BR80, BR82, *BR87*^{6B}, *BR90*^{6B}
Fibroadenosis	**BR17,** BR56
Focal duct papilloma fibrocystic	**BR66**
Periductal mastitis	**BR23**
Fibroadenomatoid change with pseudoangiomatous stromal hyperplasia	BR34
Lipoma	BR46
Fibrocystic mastopathy associated	BR51
Fibroepithelial neoplasm	*BR88*^{6B}
Sclerosing adenosis	**BR89**
Cystic papillary apocrine hyperplasia	BR29
Seromucinous cystadenoma	BR27
Focal marked squamous dysplasia with koilocytic change	BR42
Nodular goiter hyperplasia	BR57

Patient IDs in bold have more than one diagnosis and ID in italics were HHV positive, superscript indicating HHV6B or 7. *indicates samples not available for HHV DNA analysis. "a" and "b" after patient ID indicates paired samples from same patient. All samples were positive in *RNase P* PCR and negative for CuV, BuV, TuV and HHV-6A in the two multiplex PCRs.

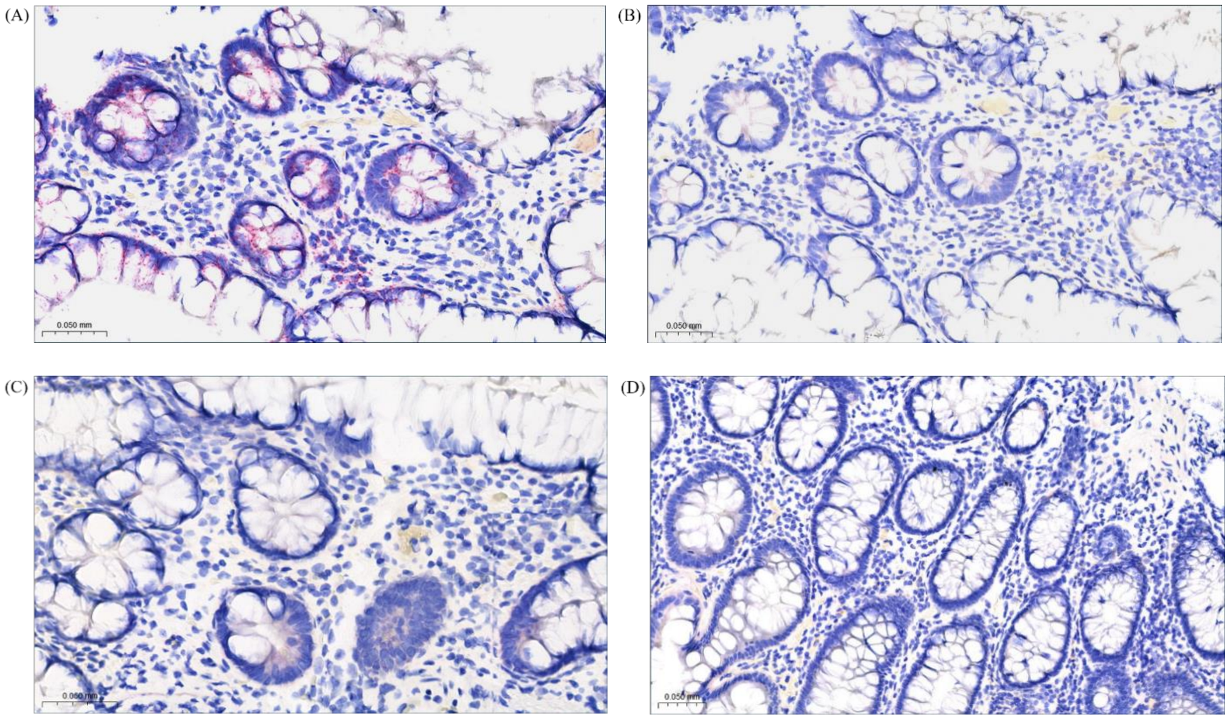

Figure S2. RNAscope ISH RED assay on healthy FFPE ileum tissues. A) human *PPIB* probe, as positive technical control and **B)** bacterial *DapB* probe as negative technical control. **C)** CuV-*NS1* probe on a CuV PCR-negative ileum from patient IBD118, as a negative biological control, and **D)** CuV-*NS1* probe on a CuV PCR-positive healthy ileum from patient IBD24, as a representative of a CuV PCR-positive but RISH-negative result. All sections counterstained with hematoxylin. Representative positive signals appear as red punctate dots, as seen in Figure A. Scale bars, 50 µm. Of note, we had no CuV RISH-positive gut tissues, but examples of CuV RISH-positive skin tissues can be found in our earlier reports [1,12].

References

1. Mohanraj U, Väkevä L, Ranki A, Söderlund-Venermo M. Prevalence, tropism, and activity of cutavirus in circulating blood lymphocytes, stool, and skin biopsy specimens of patients with cutaneous T-cell lymphoma and parapsoriasis en plaques. J Med Virol. 77:987-990, 2024; doi: 10.1002/jmv.29575.

2. Väisänen E, Fu Y, Koskenmies S, Fyhrquist N, Wang Y, Keinonen A, Mäkisalo H, Väkevä L, Pitkänen S, Ranki A, Hedman K, Söderlund-Venermo M. Cutavirus DNA in malignant and nonmalignant skin of cutaneous T-cell lymphoma and organ transplant patients but not of healthy adults. Clin Infect Dis. 68:1904-1910, 2019; doi: 10.1093/cid/ciy806.

3. McNees AL, White ZS, Zanwar P, Vilchez RA, Butel JS. Specific and quantitative detection of human polyomaviruses BKV, JCV, and SV40 by real time PCR. J Clin Virol. 34:52-62 2005; doi: 10.1016/j.jcv.2004.12.018.

4. Toppinen M, Norja P, Aaltonen LM, Wessberg S, Hedman L, Söderlund-Venermo M, Hedman K. A new quantitative PCR for human parvovirus B19 genotypes. J Virol Methods. 218:40-45, 2015.

5. Sadeghi M, Aaltonen LM, Hedman L, Chen T, Söderlund-Venermo M, Hedman K. Detection of TS polyomavirus DNA in tonsillar tissues of children and adults: evidence for site of viral latency. J Clin Virol. 59:55-58, 2014.

6. Xu M, Arku B, Jartti T, Koskinen J, Peltola V, Hedman K, Söderlund-Venermo M. Comparative diagnosis of human bocavirus 1 respiratory infection with messenger RNA reverse-transcription polymerase chain reaction (PCR), DNA quantitative PCR, and serology. J Infect Dis. 215:1551-1557, 2017; doi: 10.1093/infdis/jix169.

7. Pyöriä L, Jokinen M, Toppinen M, Salminen H, Vuorinen T, Hukkanen V, Schmotz C, Elbasani E, Ojala PM, Hedman K, Välimaa H, Perdomo MF. HERQ-9 Is a new multiplex PCR for differentiation and quantification of all nine human herpesviruses. mSphere 5: e00265-20, 2020; doi: 10.1128/mSphere.00265-20.

8. Tamura K, Nei M. Estimation of the number of nucleotide substitutions in the control region of mitochondrial DNA in humans and chimpanzees. Mol Biol Evol. 10:512-526, 1993; doi: 10.1093/oxfordjournals.molbev.a040023.

9. Tamura K, Stecher G, Kumar S. MEGA11: Molecular Evolutionary Genetics Analysis Version 11. Mol Biol Evol. 38:3022–3027, 2021; doi: 10.1093/molbev/msab120.

10. Väisänen E, Paloniemi M, Kuisma I, Lithovius V, Kumar A, Franssila R, Ahmed K, Delwart E, Vesikari T, Hedman K, Söderlund-Venermo M. Epidemiology of two human protoparvoviruses, bufavirus and tusavirus. Sci Rep. 6:39267, 2016: doi: 10.1038/srep39267.

11. Chesnut SK, Mohanraj U, Rayamajhi Thapa R, Jalilian FA, Amini R, Sedighi I, Sedighi P, Al-Hello H, Barakat AM, Masika M, Mwaengo D, Anzala O, Nora-Krukle Z, Vilmane A, Ziemele I, Manaresi E, Gallinella G, Viikari L, Jartti T, Söderlund-Venermo M. In search of human protoparvovirus acute infections. Virology. 608:110529, 2025; doi: 10.1016/j.virol.2025.110529.

12. Mohanraj U, Konttinen T, Salava A, Väkevä L, Ranki A, Söderlund-Venermo M. Significant association of cutavirus with parapsoriasis en plaques: high prevalence both in skin swab and biopsy samples. Clin Infect Dis. 77:987-990, 2023; doi: 10.1093/cid/ciad3.
